# Effect of Chemical Treatment of Sugar Palm Fibre on Rheological and Thermal Properties of the PLA Composites Filament for FDM 3D Printing

**DOI:** 10.3390/ma15228082

**Published:** 2022-11-15

**Authors:** Mohd Hakim Mohd Nasir, Mastura Mohammad Taha, Nadlene Razali, Rushdan Ahmad Ilyas, Victor Feizal Knight, Mohd Nor Faiz Norrrahim

**Affiliations:** 1 Faculty of Mechanical Engineering, Universiti Teknikal Malaysia Melaka, Hang Tuah Jaya, Durian Tunggal 76100, Malaysia; 2 Faculty of Mechanical and Manufacturing Engineering Technology, Universiti Teknikal Malaysia Melaka, Hang Tuah Jaya, Durian Tunggal 76100, Malaysia; 3 Faculty of Chemical and Energy Engineering, Universiti Teknologi Malaysia (UTM), Johor Bahru 81310, Malaysia; 4 Centre for Advanced Composite Materials, Universiti Teknologi Malaysia (UTM), Johor Bahru 81310, Malaysia; 5 Institute of Tropical Forest and Forest Products (INTROP), Universiti Putra Malaysia, Serdang 43400, Malaysia; 6 Research Centre for Chemical Defence, Universiti Pertahanan Nasional Malaysia, Kem Perdana Sungai Besi, Kuala Lumpur 57000, Malaysia

**Keywords:** sugar palm fibre, fibre treatment, cellulose, rheological properties, thermal properties, fused deposition modelling (FDM)

## Abstract

The thermal and rheological properties of bio-composite filament materials are crucial characteristics in the development of a bio-composite Fused Deposition Modeling (FDM) filament since the printing mechanism of FDM strongly depends on the heating and extrusion process. The effect of chemical treatment on the thermal and rheological properties was investigated to develop composite filaments for FDM using natural fibres such as sugar palm fibre (SPF). SPF underwent alkaline and silane treatment processes before being reinforced with PLA for improving adhesion and removing impurities. Thermogravimetric Analysis (TGA), Differential Scanning Calorimetric (DSC), and Melt Flow Index (MFI) analyses were conducted to identify the differences in thermal properties. Meanwhile, a rheological test was conducted to investigate the shear stress and its viscosity. The TGA test shows that the SPF/PLA composite treated with NaOH and silane showed good thermal stability at 789.5 °C with 0.4% final residue. The DSC results indicate that the melting temperature of all samples is slightly the same at 155 °C (in the range of 1 °C), showing that the treatment does not interfere with the melting temperature of the SPF/PLA composite. Thus, the untreated SPF/PLA composite showed the highest degradation temperature, which was 383.2 °C. The SPF/PLA composite treated with NaOH and silane demonstrated the highest melt flow index of 17.6 g/min. In conclusion, these findings offer a reference point for determining the filament extrusion and printability of SPF/PLA composite filaments.

## 1. Introduction

The need for a better, safer, and cleaner environment has awakened people’s concern about reducing the consumption of petroleum and its by-products. Recently, natural fibres have been suggested to be employed as a potential reinforcement material with comparable qualities to synthetic fibres. The increasing popularity of polymer composites reinforced with natural fibres can be attributed to their numerous advantages and remarkable features [1,2,3]. Natural fibres are divided into three categories; animal fibres such as wool, hair, and hydroxyapatite [4]; plant fibres such as bamboo, wood, corn starch [5], and sugar palm fibre; and mineral fibres such as asbestos. Natural fibre polymer composites have various advantages compared with synthetic composites in terms of biodegradability, cost, low density, and environmental friendliness [1,6,7]. They have been utilized extensively in food packaging [8,9,10,11,12], membranes [3,13], drug delivery [14], automobiles [15], aircraft interiors [16], and architectural structural applications.

**Figure 1:**
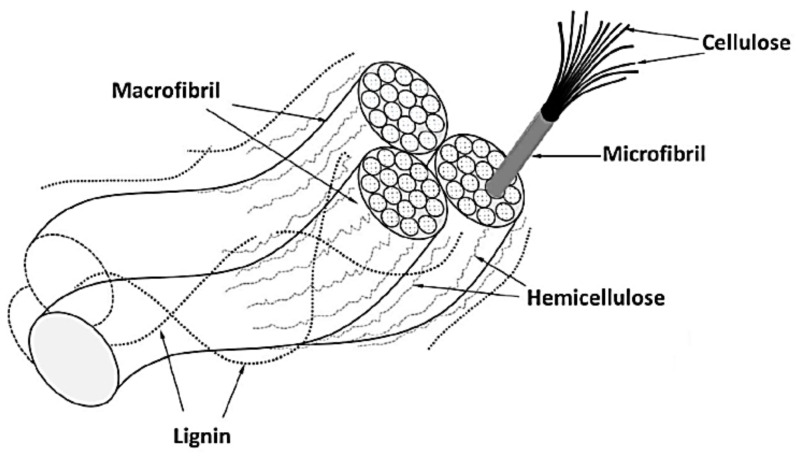
Compositions of natural fibre.

Natural fibres’ chemical composition, such as sugar palm fibre, is composed of cellulose, hemicellulose, and lignin [17]. The alignment of the cellulose fibrils, which are composed of chained-cellulose molecules, along the length of the fibre provides greater mechanical (tensile and flexural) strength and stiffness. Hemicellulose is accountable for the absorption of moisture and water, biodegradation, thermal degradation, and fibre disintegration. The cross-linking structure of lignin or pectin, which is found in plant fibres, is responsible for ultraviolet (UV) resistance and thermal stability. A schematic diagram of natural fibres’ composition is given in Figure 1 [18]. However, some issues are raised when employing plant fibres in polymers, such as destitute thermal stability and poor interfacial adhesion between the polymers and plant fibres. The absorption of moisture inside natural fibres leads to dimensional changes in the composite and loosens the interfacial adhesion. This issue was caused by the hydrophilic nature of natural fibres and the hydrophobic nature of the polymer matrix, which might lead to interfacial incompatibilities and wettability. By eliminating hemicellulose, lignin, pectin, and wax, chemically treated fibres gain surface roughness improvement. In addition, the combination of alkaline and silane treatment enhanced the density of the cellulose portion, resulting in a trans-crystalline interphase zone containing tiny crystals. The fibre was able to effectively surround and adhere to the matrix because the adhesive bond between the fibre and matrix was stronger than it was in the untreated fibre composites [19,20]. An efficient surface for fibre–matrix adhesion was formed by the chemical treatment of the surface fibre, which exposed the fibrils and provided an uneven surface topography.

Rheology is the study of material flow and deformation brought on and controlled by external forces. In the polymer industry, it is generally concerned with melt flow characteristics during polymer processing. The polymer behaves entirely differently from other materials because of its chain-like structure. Vacuum forming, blow moulding, injection moulding, extrusion, and additive manufacturing (AM) techniques depend on viscosity. Despite the rapid development of biocomposite filament for Fused Deposition Modeling (FDM), information about sugar palm fibre (SPF) composite filament is limited, mostly due to a lack of reliable data on the processability of SPF for the FDM technology. There is a need for techniques that enable the effective optimization of the material’s composition and properties, overcoming the drawbacks associated with ineffective and time-consuming trial-and-error procedures, to increase the portfolio of SPF nanoparticle-based formulations suitable for 3D-printing applications. The process for determining the FDM processability of polymeric materials must consider the polymer’s rheological characteristics and the associated critical implications during all stages of a typical FDM process because the processability of the material is strictly governed by its rheological behavior. The material must meet certain requirements to be classified as “FDM printable”. First, the polymer composite filament must be able to be extruded through the printing nozzle, with viscosity values low enough to ensure the melt’s fluidity. Second, during the deposition step, when the melt exits the nozzle, the extruded material must remain in good shape under gravity and stresses generated by the deposition of subsequent layers. Thus, a marked shear thinning behavior involving a sudden increase in the melt viscosity with a decrease in the shear rate is required to prevent shape instability and defect formation. Finally, in the post-deposition phase, polymer chains lying at the interface between adjacently deposited layers must be capable of interdiffusion across the interface to ensure a high level of interlayer adhesion and, hence, the adequate mechanical performance of the printed part [21].

**Figure 2:**
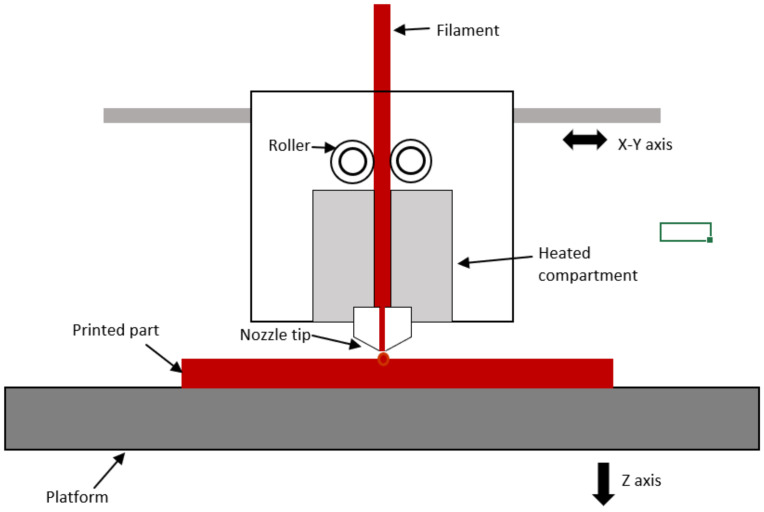
Schematic of FDM.

Fused Deposition Modeling (FDM) has been applied in additive manufacturing (AM) technologies because of its operational flexibility, time efficiency, and low cost. The technique is also known as fused filament fabrication (FFF), currently the most common 3D printing technique [14,22,23,24]. The ability to construct three-dimensional parts by FDM creates three-dimensional objects by stacking layers of thermoplastic materials with one another. A schematic diagram of FDM can be seen in Figure 2. The filament is pulled through rollers and placed into a heated compartment as the feedstock material. The filament then melts in the heated compartment and extrudes through an extrusion nozzle onto the preheated bed. The construction of the part is organized by the software that gives the coordinates to move into the x, y, and z, plane accordingly. During the printing process, the extruder subsequently moves up into the z-plane to build a layer on top of the constructed layer until a three-dimensional part is fully printed. While depositing the sequential layers, the molten layers of extruded material called lamina form bonds among the adjacent [25].

Two separate nozzles are used for the model material and support material. The support materials are water-soluble and insoluble. The finished part can easily separate by immersing the part prototyped by FDM, which contains the model and support material, into the water or chemical solution inside the tank based on the types of support materials applied. Optimization of a few critical parameters may result in cost-effective improvements in the mechanical and physical properties, as well as the dimensional accuracy of functioning 3D-printed parts [22].

During the FDM process, the thermoplastic filament is melted in a heated compartment and pushed out from the nozzle as molten threads. Thermoplastic is suitable for filament FDM because it carries the appropriate flexural strength and modulus which means that filament can be spooled. It can be heated into a low viscosity melt that can be extruded through a nozzle and subsequently becomes firm when cooling which provides an accurate dimensional of the printed part. To be a suitable material for FDM, the rheological behavior during extrusion is crucial. The melting filament cannot maintain its shape if the material solidifies too slowly. Furthermore, large stress can occur if the material solidifies prematurely and contracts significantly before taking its shape. Adhesion of the melt to the solidified printing layer is also one of the crucial criteria for an FDM filament material. The use of thermoplastic biocomposite filaments in FDM is intriguing for reducing material costs and the environmental impact, minimizing deformation during processing and possibly conserving the material’s mechanical qualities [26]. Numerous forms of bio-composite filament have been developed because of the versatility of natural composites in a variety of applications. Among the polymer matrix, PLA has been adopted to be employed with other fillers. As shown by the list in Table 1, cork, bamboo, hemp, kenaf, flax, and walnut shell are all plant fibres that have been developed by the researchers to be employed with PLA. Besides plant fibres, fibres from bone, namely hydroxyapatite (HA), also have been employed with PLA to fulfill the demand for biocomposites in the medical field.

In this paper, the authors focused on the treatment of sugar palm fibre which undergoes treatments such as alkaline (6% NaOH) solution, 2% silane solution, and a combination of 6% NaOH and 2% silane solution to modify the surface characterization of the sugar palm fibre. This paper aims to investigate the effect of chemical treatment on the thermal and rheological properties of the SPF/PLA composite filament for FDM. The effect of three different treatments of SPF/PLA for the FDM filament composite has also been studied. Recently, no studies prepared by other researchers related to SPF/PLA composite filaments for FDM have been reported.

## 2. Materials and Method

**Figure 3:**
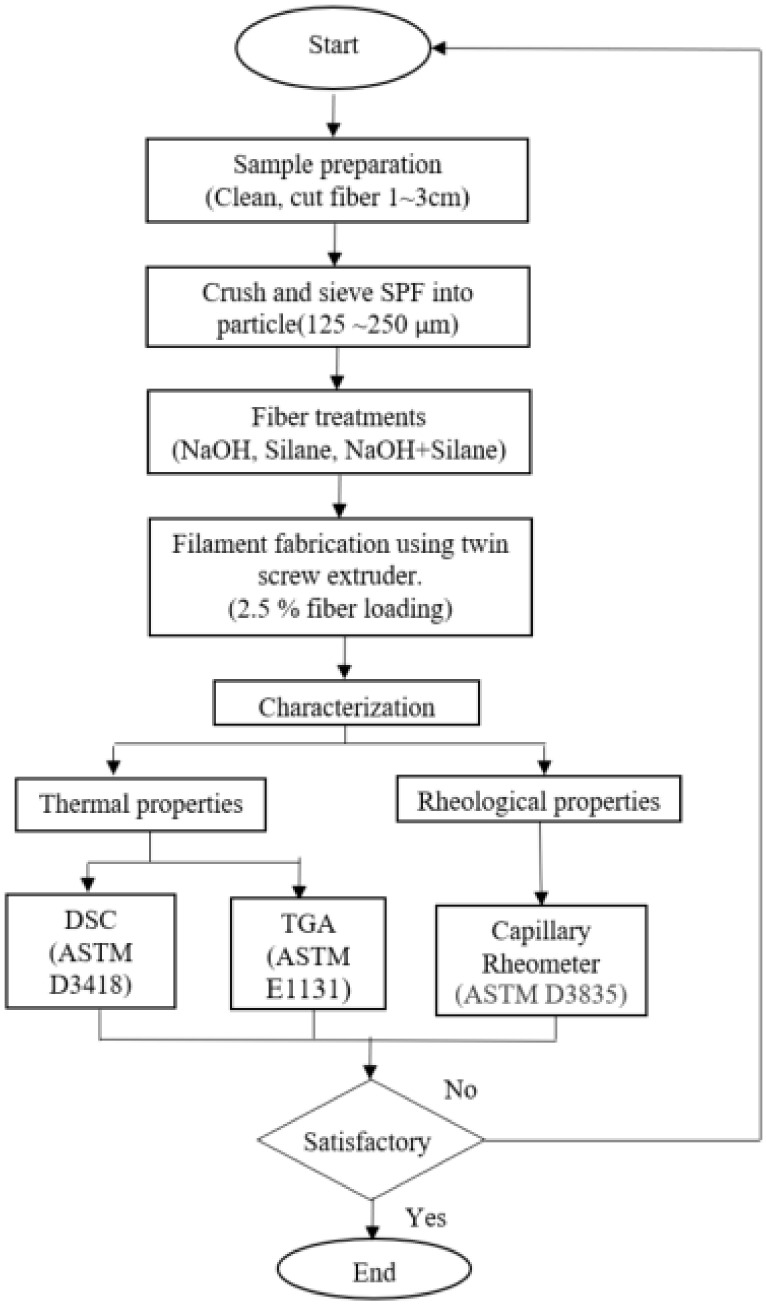
Flowchart of process methodology.

The executions of thermal and rheological experiments provide significant data regarding the determination of employing SPF/PLA composite filaments. Through those experimental studies, the selection of an optimum fibre treatment would enable the authors to decide which procedure is the best method before fabricating the filaments. Hence, the study of the whole process of the experiment is shown in the flowchart in Figure 3.

### 2.1. Materials

Sugar palm fibre (Arenga Pinnata) was obtained from rainforests located in the western peninsular of Malaysia, Jempol, Negeri Sembilan. The polymer matrix is used as polylactic acid (PLA) with a density of 1.24 g/cm^3^. Ingeo™ Biopolymer 2003D pellets (100% PLA pure) were supplied by Mecha Solve Engineering. Acetic acid (glacial), methanol, sodium hydroxide pellets for analysis EMSURE^®^, and (3-Aminopropyl) triethoxysilane were obtained from Polyscientific Enterprise Sdn. Bhd. (Melaka, Malaysia).

### 2.2. Sample Preparation

SPF was extracted from sugar palm trees. The long fibre was cut into 1~3 cm lengths and washed using tap water to remove impurities. The SPF was kept in the open air and then dried in an air-circulating oven at 60 °C for 48 h before being crushed using a high-speed crusher. An industrial sieving machine was used to obtain a particle fibre with an average size of 125~250 μm.

#### 2.2.1. Chemical Treatments

The execution of chemical treatment on SPF was to enhance the bonding between the fibre and matrix of the SPF/PLA composite. There were three (3) treatments that were executed, which are alkaline, silane, and a combination of alkaline and silane treatments.

During alkaline treatment, sodium hydroxide pellets for analysis EMSURE^®^ were used for the treatment procedure. The 6 wt.% alkaline solution was prepared. The SPF particles were immersed in the solution for 3 h at room temperature. Then, the SPF was rinsed using distilled water until a neutral pH was indicated. The fibres were dried using an oven for 48 h at 60 °C.

(3-Aminopropyl) triethoxysilane was used for silane treatment. The SPF particles were immersed in 2 wt.% silane solution for 3 h. To prepare a 2 wt.% silane solution, 3-aminopropyl-tri ethoxy silane (APS) was mixed with a mixture of methanol and distilled water (90/10 *w*/*w*). The PH of the solution was adjusted to 3.5 using acetic acid and stirred continuously for 10 min. The SPF was immersed in the solution for 3 h. Then, it was rinsed using distilled water until it became neutral. Then, the SPF was oven-dried at 60 °C for 72 h to prevent the existence of moisture.

Meanwhile, for the combination of alkaline and silane treatment, the SPF was treated with a 6 wt.% alkaline treatment (NaOH solution) and continued with a 2 wt.% silane treatment (silane solution) for the second procedure.

#### 2.2.2. Sample Listing

The list of samples to be tested for TGA, DSC, and the rheological test was decided before the extrusion process. One hundred percent PLA and untreated SPF/PLA were also prepared as an indicator for different treatments of the 2.5% SPF/PLA composite. The samples were prepared accordingly, as shown in Table 2 below. 

#### 2.2.3. Extrusion of SPF/PLA Composites

The fibre volume fraction is used to determine the weight composition of the SPF/PLA composite before the extrusion process. Generally, the fibre volume fraction is calculated according to ASTM D2584 as:

$$V_{f}=\left[\rho_{m}\;.\;w_{f}/\left(\rho_{m}\;.\;w_{f}+\rho_{f}\;.\;w_{m}\right)\right]$$

where $V_{f}$ is the volume fraction of the fibres, $w_{f}$ is the weight of the fibres, $w_{m}$ is the weight of the matrix, $\rho_{f}$ is the density of the fibres, $\rho_{m}$ is the density of the matrix [42]. The value of 2.5% is fibre loading using the volume fraction equation. The value of SPF density is 1.21 g/cm^3^, while the value of PLA density is 1.25 g/cm^3^. The 2.5% SPF/PLA composite was prepared accordingly. For each 500 g of 2.5%, the SPF/PLA composite equals 12.1 g of SPF and 487.8 g of PLA pellets.

**Figure 4:**
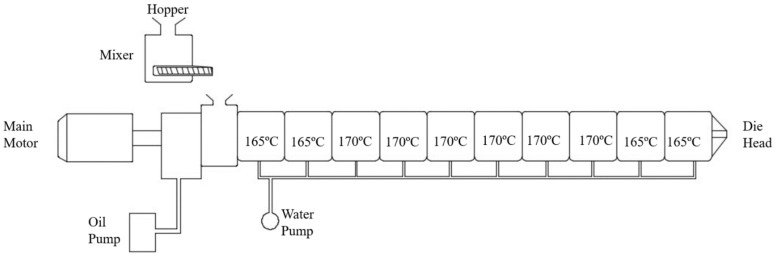
A schematic diagram of a twin screw extruder.

A twin screw extruder with 26 mm twin screws, co-rotating, 40:1 L/D from Lab Tab Engineering Company Ltd., Muang, Samutprakarn, Thailand was used for the extrusion process with 70 rpm screw speed. All the samples of the SPF/PLA composite were put into a barrel of a twin screw extruder. The mixing mechanism before the entrance of the twin screw enabled the SPF particles and PLA pellets to mix well and be conveyed into the melting compartment and extruded through a die, as demonstrated in Figure 4.

### 2.3. Thermogravimetric Analyzer (TGA) Test

The instrument used for the TGA test was a Mettler Toledo TGA. The temperature for the samples was 30 to 800 °C with a heating rate of 20 °C/min, and the atmosphere used was nitrogen gas. The nitrogen flow was 50 mL/min. All the granules of 2.5% SPF/PLA composites were weighed to be between 15–20 mg and placed into the chamber. The TGA test was conducted to measure the change in the mass of the sample as the temperature increased and the final residue yield brought about by the degradation of the SPF/PLA composite. The TGA curve graph was a plot of the weight percentage against temperature.

### 2.4. Differential Scanning Calorimetry (DSC)

Differential scanning calorimetry (DSC) analysis was performed with a heating rate of 20 °C/min, from 30 to 800 °C. The differential scanning calorimetry (DSC) result was obtained using DSC Q20 V24.11 Build 124. Mettler Toledo.

### 2.5. Melt Flow Index (MFI)

The MFI was determined using a melt flow indexer (Ray Ran- 6MPCA advanced melt flow system). The melt flow properties of the SPF/PLA composites were measured at a temperature of 190 °C with a weight loading weight of 2.16 kg. All the samples were measured for a weight of 12 g. Then, 300 s of pre-heating was conducted when the samples were fed into the instrument’s chamber. The test result was an average value obtained from the testing of two specimens.

### 2.6. Rheology

The rheological measurements were taken using an Instron capillary rheometer modelSR20 (Instron, Norwood, MA, USA) at different piston speeds in the range of 0.00024–1200 mm/min. The capillary used was made of tungsten carbide with a length-to-diameter (L/D) ratio of 5:1. The samples with 0, 2.5, 5, 7.5, and 10 wt.% SPF/PLA composite was loaded into the barrel of the extrusion assembly and forced down into the capillary using a plunger. The experiment began by setting the die temperature to 175 °C, followed by 185 °C and 195 °C for each sample. The shear rate values were set from 200, 400, 600, 800, and 1000 s^−1^. After allowing a resting time of 5 min, the melt was extruded through the capillary at predetermined plunger speeds.

## 3. Results and Discussion

### 3.1. Thermogravimetric Analyzer (TGA) Test

**Figure 5:**
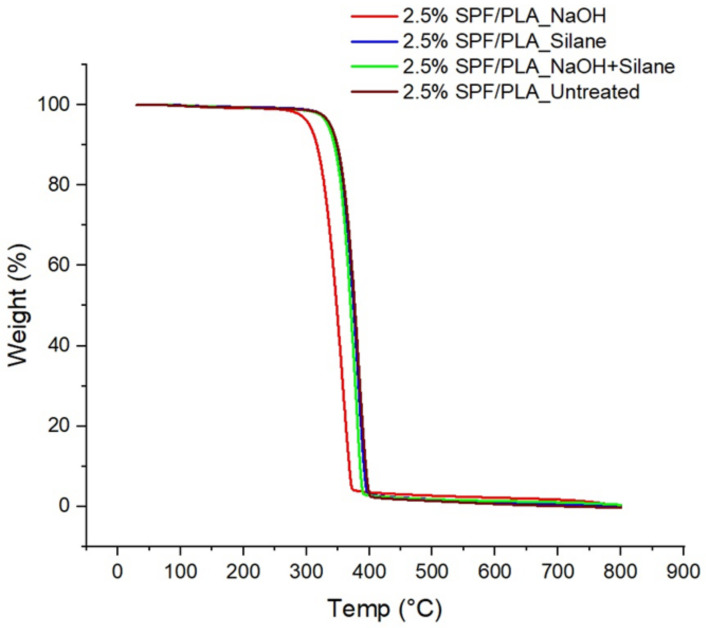
TGA results of 2.5% SPF/PLA filament composites.

Thermogravimetric analysis is important to determine the thermal stability and kinetic parameters of the SPF/PLA composite. From the TGA data, we can determine the thermal stability and heat resistance of the composite. It is important to investigate the degradation and decomposition of the composite under certain heat exposure to optimize the extrusion capabilities during any application [43] and identify the effect of different chemical treatments. TGA curves of filaments with untreated and treated fibres are shown in Figure 5. The TGA curve starts with the evaporation of water molecules, decomposition of lignocellulosic, cellulose, and lignin, and final residue [44].

From the results in Table 3, the initiation temperature is the lowest for the untreated composite, which begins at 118.9 and 119.8 °C for the NaOH-treated composite, 127.8 °C for the silane-treated composite, and 133.6 °C for the NaOH- and silane-treated composite. The process resulted in the lowest temperature for the untreated fibre. Since the fibre is hydrophilic, it already absorbs water, and the lowest temperature in the initial stage can be attributed to the loss of water that has been absorbed. According to Sanyang [45] and Ilyas [46,47,48], the mass loss during this stage can be related to the evaporation or dehydration of poorly-bound water and the low molecular weight compound in the mixed nano bio-composite. Due to the presence of –OH functional groups, various cellulose-based fibres exhibit this general characteristic [49]. Atiqah et al. [50] reported the initiation temperatures for the first stage to be around 132 °C and 156 °C for sugar palm fibre/glass with TPU composite.

In the second stage, the decomposition of the SPF/PLA composite began at temperatures of 200–400 °C, as hemicellulose, cellulose, pectin, and lignin started to decompose. This process was initiated at 356.6 °C for the NaOH-treated composite, 375.2 °C for the NaOH and silane-treated composite, 377.8 °C for the silane-treated composite, and the highest temperature of 379.7 °C was recorded for the untreated composite. The drastic weight reduction that occurred for all of the samples indicated the highest thermal degradation beyond 300 °C.

### 3.2. Differential Scanning Calorimetry (DSC)

**Figure 6:**
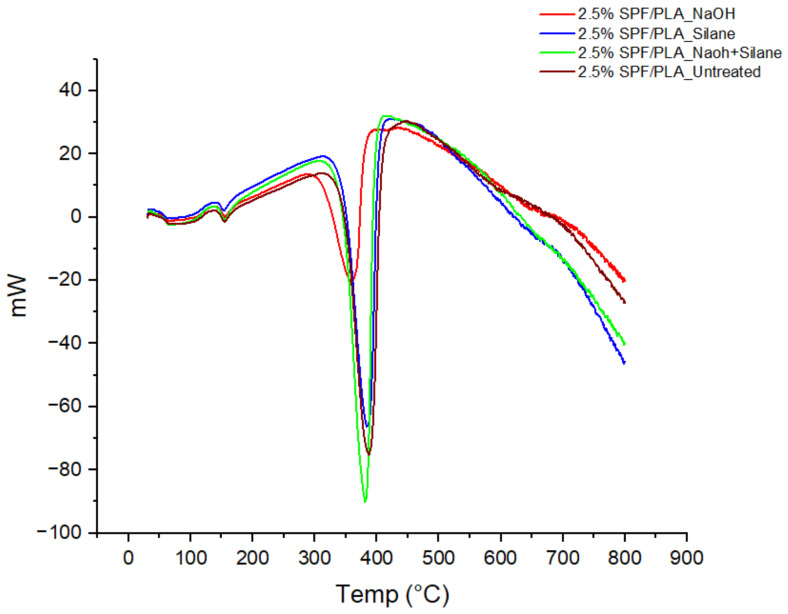
DSC results of 2.5% SPF/PLA filament composites.

Differential scanning calorimetry (DSC) analysis was performed to understand the thermal properties of the SPF/PLA composite filament. It is a very useful method for studying the glass temperature (T_g_), degradation temperature, and melting behavior of composites. The T_g_ of a composite relies on its molecular properties, content, and compatibility [28]. The DSC analysis shows the T_g_, melting temperature (T_m_) and degradation temperature data of the treated NaOH, treated silane, treated NaOH + silane, and untreated SPF/PLA composite. From the results obtained as listed in Table 4, the T_g_ temperature of the SPF/PLA composite was affected by the chemical treatment of SPF. The glass transition temperature T_g_ depends on the molecular characteristics, composition, and compatibility of the components in the composite [28]. Poor compatibility with added fillers can decrease the T_g_ [51]. Therefore, as seen in Figure 6, the lowest T_g_ of the SPF/PLA composite was from the untreated sample at 54.0 °C, while for the other samples, the results were 60.9 °C for the treated NaOH, 54.9 °C for the treated silane, and 58.9 °C for the treated NaOH and silane, as seen in Figure 6. This can be described as a weak interfacial adhesion between the untreated SPF and PLA due to poor compatibility. The presence of untreated SPF in the PLA matrix interfered with the crystallization process of the PLA.

The crystallization temperature T_c_ that has been achieved from the test is 137.7 °C for untreated composite, 139.0, 137.9, and 137.7 °C for the NaOH, silane, and NaOH + silane treated SPF/PLA composites, respectively. It seems that there was no significant effect of Tc for the SPF/PLA composite filament. The melting temperature of all the SPF/PLA composites showed no significant effect for the chemically treated or untreated SPF/PLA composites, and the melting temperatures were 155.0 °C for the untreated SPF, 155.1 °C for NaOH, and 154.3 °C for the silane-treated SPF and NaOH as well as the silane-treated SPF PLA composite. The results indicate that the T_m_ of the SPF/PLA composite is slightly the same (in the range of 1 °C), showing that the treatment does not interfere with the melting temperature for the SPF/PLA composite. This finding is similar to the finding by A. Hayati et al. [39], who found that the difference is within the range of 1 °C (149.6~150.9 °C ) for the melting temperature of chemically-treated kenaf fibre/PLA composites.

However, the degradation temperature for all the samples of the SPF/PLA composite showed an effect of chemical treatment. The highest degradation temperature among those samples was that of the untreated SPF/PLA composite which was 383.2 °C, followed by 358.4 °C for the NaOH treated composite, 376.1 °C for the NaOH and silane treated composite, and 380.3 °C for the silane-treated SPF/PLA composite. This indicates that the untreated SPF contains a variety of substances, including cellulose, hemicellulose, and other substances that had to be burned, thus raising the melting point and reducing crystallization enthalpy and crystallinity [28].

### 3.3. Melt Flow Index (MFI)

**Figure 7:**
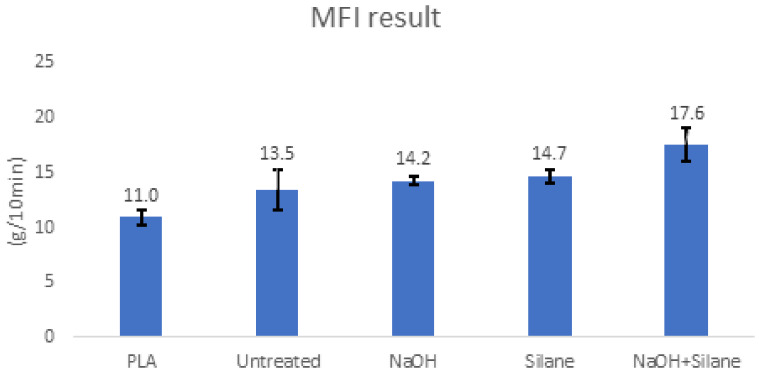
The 2.5% SPF/PLA composite MFI result.

Evaluation of the MFI helps to determine how fibre treatment would affect SPF/PLA’s processability for 3D printing. The value of MFI determines how a polymer material flows in the molten state and is inversely proportional to the dynamic viscosity. It affects the polymer processability and is well linked to the adhesion between layers of printed parts. In terms of PLA, an MFI value above 10 g (10 min)^−1^ [52] is the minimum value needed to ensure good flow properties with no clogging at the nozzle during printing. Pure PLA filament has also been tested as a benchmark for the employment of different chemically treated SPFs. The reduction of MFI and, thus, the increase in the viscosity of the biocomposite materials was related to the reduction in polymer chain mobility [53]. Figure 7 revealed that the presence of SPF particles in the PLA matrix increased the flow rate of the polymer composite compared with neat PLA. The combination of alkaline and silane treatment showed the highest flow rate of 17.64 g/min, compared to untreated SPF with a flow rate of 13.48 g/min. As from the data received, the largest standard deviation was observed in the untreated composite with a value of 1.8 and the smallest standard deviation was from the NaOH treated composite with a value of 0.4. The higher MFI value was probably due to a significant amount of auto-polymerization silane molecules, which contain lighter-weight molecules, resulting in the easy flow of biocomposites [41]. This finding is similar to those of Moustafa et al. [54], who found that the MFI of spent coffee ground (SCG) filler increased respectively with the amount of SCG content. The combination of alkaline and silane treatment, as expected, eliminated all impurities and unwanted substances (hemicellulose, lignin, and pectin), which led to greater bonding with the PLA matrix. The flow was smoother, and MFI was higher compared to untreated fibre, in which the fibre and polymer were poorly bonded, resulting in a slower flow rate [55]. Similar results were also reported by Eselini et al. [56], who discovered that the MFI of the basal, flax/PLA composite was larger than the PLA. The MFI values were increased by the incorporation of basalt fibre (BF) and flax fibre (FF) into the PLA matrix. During the processing of composites, the increased amount of fibre added to PLA increases the shear stress. This may account for the increase in the MFI value of PLA after fibre incorporation. At the same fibre content and coupling agent levels, the MFI of the treated fibre composite was higher than the untreated fibre composite.

### 3.4. Rheology

**Figure 8:**
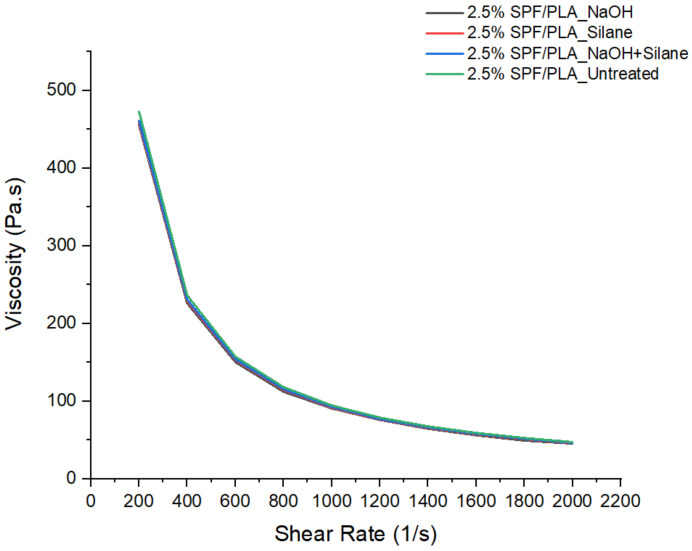
Results of viscosity vs. shear rate.

The purpose of performing the rheological test was to determine how the stress in a material or force applied is related to the deformation and flow of the sugar palm fibre polymer composite. The Instron SR20 was used to identify the rheological properties of the samples. Rheological tests were successfully performed starting at a temperature of 175 °C. At this temperature, the composite material was able to flow out through the nozzle. As a result, this is the recommended minimum temperature for the printing process of FDM. However, during a trial of printing the composite filament using a 3D printer, it was found that the suitable temperature for the nozzle setting was in the range of 190~200 °C, slightly higher than the set temperature of the rheological test. As observed, there were no significant differences in viscosities curves between the untreated SPF/PLA composite and the chemically treated NaOH and the silane SPF/PLA composites. Those curves are typically shear thinning, thus demonstrating the non-Newtonian behavior of fluids whereby the viscosity decreases under shear rate. It is synonymous with pseudoplastic behavior [57], which is mainly influenced by the orientation of polymer molecules, the agglomeration of sugar palm fibre, and entanglements within the PLA chains in the capillary rheometer. The agglomeration increased the viscosity as the fibres intertwined in the composites. Thus, it prevented the flow of the matrix through the composite. The difference in the curve might occur if the SPF content is not fixed or higher than 2.5% fibre content. Mazzanti et al. [58] found that the higher hemp fibre content in the PLA matrix shows a significant drop in the viscosity curve. The very dilute nanocomposites such as the SPF/PLA composite only caused a negligible increase in the viscoelastic function concerning the pure matrix because of the hydrodynamic interactions among the particles. As seen in Figure 8, the same curve pattern between the untreated and chemically-treated SPF/PLA is similar to the finding by Durmus [59] who concluded that the employment of an adhesion promoter or a compatibilizer in wood polymer composite (WPC) composition and its amount and specification are more important parameters than the size of the wood particle and the chemical treatment which enhanced the interfacial interactions between the PP and the wood fibre.

## 4. Conclusions

In this study, the thermal and rheological properties of different treatment SPF/PLA composite filaments were analyzed, in order to propose the best treatment of SPF suitable for FDM composite materials. FDM involved a variety of heating processes, such as extrusion and printing, demanding knowledge of the thermal characteristics and rheological behavior of the materials used. Fabricating an FDM filament from an SPF and PLA composite provides another option for a composite filament that suits the demand in the fields of bio-medical appliances, the automotive industry, the aerospace industry, and household appliances. TGA and DSC studies aid in determining the heating parameters used for the extrusion process during extrusion and printing. The treated filament with NaOH and silane showed better thermal degradation and thermal stability than the other treated filaments with 133.57 °C at the early stage and 789.54 °C for the final residue. Furthermore, in the DSC result, the untreated composite filament also showed the lowest T_g_ and the highest T_m_ among the other treated composite filaments indicating that the chemical treatment affected those results. The treatment with the combination of NaOH and silane also showed the highest flow rate in MFI (17.64 g/min). However, there was no significant effect of chemical treatment in terms of the rheological test. Summing up, the presented results are encouraging for the development of biodegradable filament composites, as they could aid in reducing the environmental consequences. Further investigations into the effects of chemical treatment on mechanical and physical properties need to be carried out to find the relationship between the thermal, rheological, and mechanical properties of this filament composite.

## Figures and Tables

**Table 1:** PLA-based composite filament.

Ind.	Type of PLA Composite Filament	Reference
1	Cork/PLA	[27]
2	Wood Flour/PLA	[28,29,30,31,32]
3	Bamboo fill, Wood fill, Pine lay wood/PLA	[33]
4	Hemp/PLA	[34]
5	Hydroxyapatite (HA)/PLA	[4]
6	Cellulose Nanofibrils/PLA	[35]
7	Coffee grounds/PLA	[36]
8	Flax, bamboo/PLA	[37,38]
9	Kenaf/PLA	[39]
10	Hap-Cs/PLA	[40]
11	Walnut shell/PLA	[41]

**Table 2:** List of samples.

Ind	Type of Treatment	Name of Sample
1.	NaOH	2.5% SPF/PLA_NaOH
2.	Silane	2.5% SPF/PLA_silane
3.	NaOH + silane	2.5% SPF/PLA_NaOH + silane
4.	Untreated	2.5% SPF/PLA_untreated

**Table 3:** Results obtained from the TGA test.

Thermal Properties			
Parameter	Initiation Temp. (°C)	Max. Decomposition Temp. (°C)	Final Residue (%)
2.5% SPF/PLA_Untreated	118.9	379.7	0.3
2.5% SPF/PLA_NaOH	119.8	356.6	0.3
2.5% SPF/PLA_Silane	127.8	377.8	0.3
2.5% SPF/PLA_NaOH+Silane	133.6	375.2	0.4

**Table 4:** Results obtained from the DSC test.

	Thermal Properties			
Parameter	T_g_ (°C)	Tc (°C)	T_m_ (°C)	Degradation Temperature (°C)
2.5% SPF/PLA_Untreated	54.0	137.7	155.0	383.2
2.5% SPF/PLA_NaOH	60.9	139.0	155.1	358.4
2.5% SPF/PLA_Silane	54.9	137.9	154.3	380.3
2.5% SPF/PLA_NaOH+Silane	58.9	137.7	154.3	376.1

## Data Availability

Not applicable.
